# Establishment and assessment of rodent models of medication-related osteonecrosis of the jaw (MRONJ)

**DOI:** 10.1038/s41368-022-00182-4

**Published:** 2022-08-10

**Authors:** Ran Yan, Ruixue Jiang, Longwei Hu, Yuwei Deng, Jin Wen, Xinquan Jiang

**Affiliations:** 1 Department of Prosthodontics, Shanghai Ninth People’s Hospital, Shanghai Jiao Tong University School of Medicine, Shanghai Jiao Tong University, Shanghai, China; 2 College of Stomatology, Shanghai Jiao Tong University, Shanghai, China; 3 National Center for Stomatology, National Clinical Research Center for Oral Diseases, Shanghai Key Laboratory of Stomatology, Shanghai Engineering Research Center of Advanced Dental Technology and Materials, Shanghai, China; 4 Department of Oral and Maxillofacial-Head and Neck Oncology, Shanghai Ninth People’s Hospital, Shanghai Jiao Tong University School of Medicine, Shanghai Jiao Tong University, Shanghai, China

**Keywords:** Experimental models of disease, Preventive medicine

## Abstract

Medication-related osteonecrosis of the jaw (MRONJ) is primarily associated with administering antiresorptive or antiangiogenic drugs. Despite significant research on MRONJ, its pathogenesis and effective treatments are still not fully understood. Animal models can be used to simulate the pathophysiological features of MRONJ, serving as standardized in vivo experimental platforms to explore the pathogenesis and therapies of MRONJ. Rodent models exhibit excellent effectiveness and high reproducibility in mimicking human MRONJ, but classical methods cannot achieve a complete replica of the pathogenesis of MRONJ. Modified rodent models have been reported with improvements for better mimicking of MRONJ onset in clinic. This review summarizes representative classical and modified rodent models of MRONJ created through various combinations of systemic drug induction and local stimulation and discusses their effectiveness and efficiency. Currently, there is a lack of a unified assessment system for MRONJ models, which hinders a standard definition of MRONJ-like lesions in rodents. Therefore, this review comprehensively summarizes assessment systems based on published peer-review articles, including new approaches in gross observation, histological assessments, radiographic assessments, and serological assessments. This review can serve as a reference for model establishment and evaluation in future preclinical studies on MRONJ.

## Introduction

Medication-related osteonecrosis of the jaw (MRONJ) is an adverse side effect of antiresorptive and antiangiogenic medications widely used to treat bone metastasis and osteoporosis^1^. The clinical manifestation of MRONJ is bone exposure with or without intraoral/extraoral fistula in the maxillofacial region, lasting over 8 weeks^1,2^, which is staged 1 to 3 (stage 0 represents the prodromal period without specific clinical or radiographic symptoms) according to the MRONJ staging system updated by the American Association of Oral and Maxillofacial Surgeons (AAOMS) in 2014^1,3^. Although keeping new ossified periosteum for the neo-mandible has been utilized as a therapeutic approach, massive resection of the jaw bone caused by MRONJ still affects the physiological and mental health of patients^4^. Furthermore, the pathogenesis of MRONJ has not been fully illuminated. Previous clinical and preclinical studies have indicated that systemic risk factors (e.g., drug administration^5–9^, patients’ medical conditions^10,11^) and local oral risk factors (e.g., tooth extraction^12,13^, pulpitis^14^, and periodontitis^15^) participate jointly in the development of MRONJ.

**Fig. 1:**
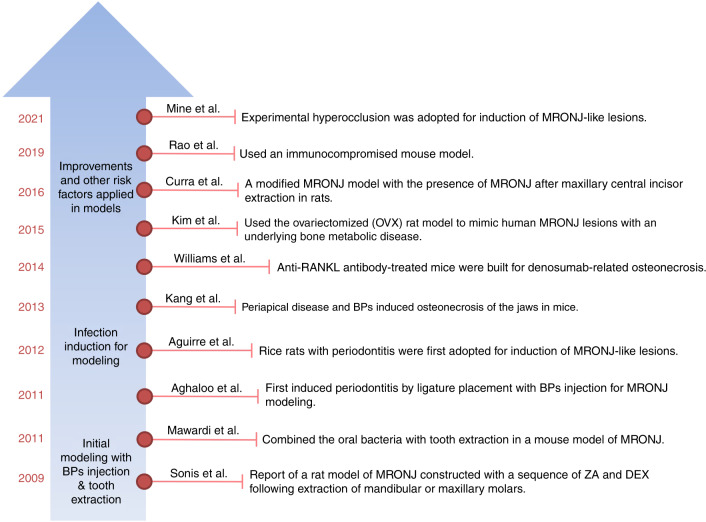
Footprints of MRONJ rodent models. Representative MRONJ rodent models from 2009 to 2021 were established by Sonis et al.^111^, Mawardi et al.^112^, Aghaloo et al.^79^, Aguirre et al.^16^, Kang et al.^41^, Williams et al.^76^, Kim et al.^26^, Curra et al.^6^, Rao et al.^81^, and Mine et al.^113^

The animal model is an essential in vivo experimental platform for exploring the pathogenesis of and interventions for various diseases. Compared with large vertebrates, rodents have universal benefits of easy feeding and operation and relatively low cost. Among all kinds of MRONJ animal models from 2003 to date, most were rodents, as 60% were rats, 27% were mice, and 2% were rice rats^16^. Large vertebrates such as dogs (~4%), pigs (~3%), and sheep (~2%) have also been applied to establish MRONJ models^16^. However, rodents boast additional advantages in MRONJ research because the presence of Haversian remodeling in large vertebrates is not involved in the development of MRONJ. Exploration into the pathogenesis of and interventions for MRONJ in rodents has been ongoing since the initial case report of MRONJ in 2003 (Fig. 1). Sonis et al. first reported an MRONJ model in rats in 2009, which was established by systemic administration of antiresorptive drug following extraction of molars^17^. In their study, rats treated with zoledronic acid (ZA), an antiresorptive drug, plus dexamethasone (DEX) presented unhealed extraction sites characterized by a base of exposed bone, some erythema, necrotic bone, and areas of inflammatory infiltration. These macroscopic and histologic findings are consistent with established criteria of MRONJ diagnosis in clinic, indicating the occurrence of MRONJ in rats. Since then, drug administration as a systemic risk factor combined with tooth extraction as a local risk factor has become the classical method of MRONJ model establishment.

Over time, modified rodent models with local inflammation and infection have been established to complement the simulation of infectious tooth extraction or spontaneous MRONJ without invasive operation in clinic. These modified MRONJ rodents were established by local risk factors of pulpitis or aggressive periodontitis^18,19^. Another powerful model for spontaneous periodontitis, the rice rat, has also been utilized in MRONJ research^20,21^. Apart from different local risk factors adopted in establishing models, to further simulate abnormal bone tissue metabolism conditions exhibited by MRONJ patients, such as osteoporosis, researchers have created MRONJ models based on ovariectomized (OVX) rodents^8,16,22–28^.

**Fig. 2:**
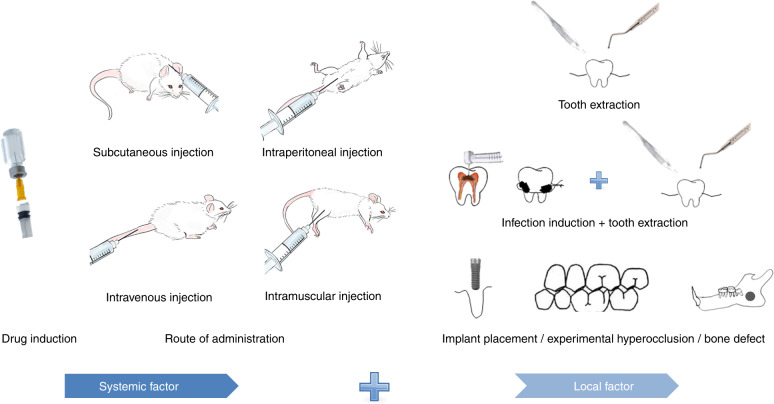
General procedure of establishing MRONJ rodent models. The first step is drug administration with bisphosphonates or other related drugs by subcutaneous injection, intraperitoneal injection, intravenous injection, or intramuscular injection. The second step is to deploy local stimulation identified as a common risk factor, such as tooth extraction, infection induction, or mechanical stimuli

In general, the approach of establishing MRONJ rodent models mainly constitutes two steps: drug administration as a systemic risk factor and oral stimulation as a local risk factor (Fig. 2). The ideal MRONJ model should imitate the pathophysiological characteristics of MRONJ, including bone necrosis, inflammatory cell infiltration, and angiogenesis inhibition. Current MRONJ models only mimic some of the pathophysiology with the systemic and local risk factors used for model establishment. At present, specific questions, such as which rodent^29,30^, drug dosage and frequency^20,31–36^, and local risk factor^18,37–42^ should be selected, have not received unitive answers yet. Thus, in this review, we summarize the selections made by existing classical and modified rodent models of MRONJ.

Furthermore, the assessment of the established MRONJ model plays a critical role in proving the effectiveness and efficiency of the methods and in verifying the reliability of the models. As various techniques have been introduced for the assessment of MRONJ models, we comprehensively overview current techniques in gross observation, histological assessments, radiographic assessments, and serological assessments, which will contribute to the establishment of a unified MRONJ model assessment system. To reduce variation in the criteria used to define MRONJ in current studies, we also summarize highlights in the assessment of MRONJ.

## Approaches of establishing MRONJ models

### Classical method

Systemic drug induction plus tooth extraction is the most popular approach adopted for MRONJ rodent model establishment, as tooth extraction is the most commonly reported local risk factor. In fact, 52%–61% of MRONJ patients have a history of tooth extraction, and an individual’s risk of MRONJ is 16 times higher after tooth extraction^43^. In previous rodent animal studies, the protocol used to establish MRONJ models includes the selection of animal, drug type, dosage, duration, co-medications, route of administration, tooth extraction site, and the time interval between tooth extraction and drug induction.

Considering those discrepancies in each part may cause diverse protocols for MRONJ models, we carefully analyzed and elucidated the most commonly used protocols, taking into consideration the animal selection, drug type, dose, administration route and time, and tooth extraction sites and intervals. Given the cost and effectiveness, we also sorted out the differences in administration times, total duration, and success rates between classical methods to provide references for subsequent researchers in establishing their protocols (Table 1).

**Table 1:** Collection of MRONJ rodent models established by the classical method

Species	Age/week	Sex	Drug	Dose /(mg·kg^−1^)	Frequency	Administration route	Induction time/week	Times of administration	Extraction site	Interval	Total duration/week	MRONJ characteristics	Success rate	Ref.
Rat	4	F	ZA + DEX	ZA: 0.066 DEX: 5	Thrice a week	SC	6	36	The right max M1	TE after 2 weeks of drug administration	6	Exposed NB, incomplete epithelial continuity; EL; bone formation↓BV/TV↓	100%	^59^
Mouse	8	F	ZA	ZA: 0.5	Weekly	SC	8	8	The right man M1, M2	TE after 2 weeks of drug administration	8	Formation of EL	ND	^114^
Nude mouse	8–10	F	mAb	mAb: 10	Thrice a week	IP	Approximately 4	12	The left max M1	TE conducted 1 week after the first antibody injection	Approximately 4	Absence of OCs and accumulation of NB	ND	^61^
Rat	12	F	ZA	ZA: 0.035	Once every 2 weeks	IV	8	4	The right man M1	TE after 3 weeks of drug administration	11	NB area↑; number of stranded OCs↑	33.3%	^44^
Rat	7	ND	ZA	ZA:0.066	Weekly	IP	1–2	1/2/3	Man M1, M2 of both sides	TE after 1 week of drug administration	Approximately 3	Alveolar mucosal defects, granulation tissue, and exposed bone; BV/TV↓; osteonecrosis and EL↑; numbers of OCs↓	ND	^97^
Rat	12	ND	ZA	ZA: 0.04 mg per rat	Weekly	TVI	5	5	The right max M1	TE on the 7th week(drug induction has finished)	15	Exposed NB	100%	^62^
Rat	10–12	F	ZA + DEX	ZA: 0.1 DEX: 1	ZA:thrice a week for 4 weeks +ZA: 4 times a week, DEX: weekly for 3 weeks	IP	7	27	The right max molars	TE on the 11th week (drug induction has finished)	14	BE; number of EL↑; number of blood vessels↓	25%	^45^
				ZA: 0.1 DEX: 1	ZA: Thrice a week for 4 weeks +ZA: 4 times a week, DEX: Weekly for 3 weeks + additional ZA for 3 weeks		10	39					50%	
Rat	8	F	ZA + DEX	ZA: 0.066 DEX: 5	Thrice a week	SC	4	24	The left max M1	TE after 2 weeks of drug administration	4/6	Exposed NB and EL; average number of OCs per linear bone perimeter↓; BV/TV, Tb.N, Tb.Th↓Tb.Sp↑	100%	^46^
Rat	8	M	ZA	ZA: 0.16	5 times at Weekly intervals	Jugular vein injection	5	25	The right max M1	TE after 3 d after the third administration	5	BE; EL	100%	^63^
Rat	12	F	ZA	ZA: 0.1	Thrice a week	IP	9	27	The left max molars	TE after 7 weeks of drug administration	11	BE without abscess nor fistula; the number of osteocytes ↓EL↑; TNF-α and IL-1β in the gingival tissue↑	91.66%	^47^
Rat	16–18	F	ZA	ZA: 0.1	Thrice a week	IP	6/7	18/21	The left man M1	TE after 3 weeks of drug administration	6/7	Abscesses with purulent content; no clear signs of bone formation; EL	ND	^55^
Rat	10	M	ZA + DEX	ZA: 0.1 DEX: 1	Twice a week	SC	6	24	The right max M1	TE after 2 weeks of drug administration	6	Unhealed oral mucosa, exposed NB, number of polymorphonuclear cells, and EL↑	66.67%	^64^
Rat	4	F	ZA + DEX	ZA: 0.066 DEX: 5	Thrice a week	Percutaneous injection	4	24	The right max M1	TE after 2 weeks of drug administration	4	Exposed NB, incomplete epithelial continuity, insufficient formation of connective tissue, and infiltration of white blood cells	100%	^60^
Rat	9	F	ZA + DEX	ZA: 0.035 DEX: 1	ZA: Weekly DEX: Everyday	ZA: TVI DEX: IP	3	24	The left max molars	TE after 3 weeks of drug administration	5/11	The socket was not covered with mucosa; exposed alveolar bone; BV/TV↓	ND	^48^
Rat	9–11	M	ALN + DEX	ALN: 0.2 DEX: 1	Everyday	SC	ALN: 2 DEX: 4d	18	The unilateral M1, M2	ALN injection once daily for 14 days, starting the day of TE, plus 1 mg·kg^−1^ DEX once daily for 4 d, starting 2 d before TE	Approximately 2	Open wounds; erythema; exposed bone; infection and osteonecrosis	84.62%	^68^
Mouse	8–10	F	ZA + DEX	ZA: 0.125 DEX: 5	Weekly	TVI	8	16	Bilateral max M1	TE after 2 weeks of drug administration	10	Inflammatory infiltration and unhealed mucosa; the NB; wound healing↓; BV/TV↓; numerous osteocytes with EL, inflammatory infiltrates, and the mucosa exposed chronically	Histopathologic: 78% Gross: 56%	^31^
Rat	4	F	ZA + DEX	ZA: 0.125 DEX: 5	ZA: Twice a week DEX: Weekly	IP	4	12	The right man M1	TE after 4 weeks of drug administration	12	Incomplete wound healing and the presence of exposed bone; BV/TV, Tb.N, BMD↓Tb.Sp↑; EL↑TRAP-positive cells↓	100%	^69^
Mouse	8–12	F	ZA + CY	ZA: 0.05 CY: 100	Twice a week	ZA: SC CY: IP	Prevention:7 Treatment:5	20/28	The max M1	TE after 3 weeks of drug administration	7/9	Bone fill↓; EL↑	ND	^115^
Mouse	8	F	ZA + CY	ZA: 0.05 CY: 150	ZA: Twice a week CY: Twice and once a week before and after tooth extraction	ZA: SC CY: IP	5/7	18/24	The max M1	TE after 3 weeks of drug administration	5/7	Exposed bone; wound open areas↑; OCs on bone surfaces of tooth extraction sockets↓; serum TRAcP5b levels↓; living bone and osteocyte density↓; NB and the number of EL↑; Tb.N, Tb.Th↓Tb.Sp↑	92.8%	
			mAb+CY	mAb: 5 CY: 150	mAb: Once every 3 weeks CY: Twice and once a week before and after tooth extraction	mAb: SC CY: IP		9/12					92.8%	^51^
			ZA/CY/mAb	ZA: 0.05/ CY: 150/ mAb: 5	ZA: Twice a week CY: Twice and once a week before and after tooth extraction mAb: Once every 3 wks	ZA: SC CY: IP mAb: SC		1/2/8/10/14					CY : 50% ZA: 0	
Rat	8	M	ZA + DEX	ZA: 0.125 DEX: 5	ZA: Twice a week DEX: Weekly	IP	5	15	The left max M1	TE after 1 week of drug administration	5	BE; soft tissue unhealed	80%	^65^
Mouse	8	F	mAb+CY	mAb: 5 CY: 150	mAb: Once every 3 weeks CY: Twice and once a week before and after tooth extraction	mAb:SC CY: IP	5/7	9/11	bilateral max M1	TE after 3 weeks of drug administration	5/7	Open wounds with BE; Tb.N, Tb.Th↓Tb.Sp, BMD↑; living bone area, osteocyte density↓; the number of EL↑	5w: 87.5%	^52^
Rat	ND	M	ZA	ZA: 0.1	Thrice a week	IP	8	24	The right max M1	TE after 1 week rest at the end of the 8th week	13/17	Newly formed bone tissue↓	ND	^56^
Rat	8-12	F	ZA	ZA: 0.1	Thrice a week	IP	9	27	The right max molars	TE on the 8th week	11	Mucosal ulcerations at the teeth extraction site, frequent exposure of NB; formation of granulation tissue, inflammatory cell infiltrates, fibrosis, and sequestra	33%(implantation of saline/β-TCP constructs)	^57^
Mouse	8	M	ZA	ZA: 0.125	Twice a week	TVI	4	8	The max M1	TE after 1 week of drug administration	5	Opened extraction site; delay in wound healing; discontinuous keratinized coverage with dead bone formation	40%	^116^
Rat	8	M	ZA	ZA: 0.04 mg per rat	Twice a week	IP + TVI	5	10	The M1 (the left or right side was randomly determined)	TE on 2 weeks after completion of the drug administration	8	Several empty bone lacunae; marginal bone loss; teeth with necrotic pulps; numerous sequestrates (NBs) with infiltration of acute and chronic inflammatory cells	87.5%	^99^
Rat	6	F	ZA	ZA: 2.25	Everyday	IP	3	21	The left max molars	TE after 3 weeks of drug administration	Approximately 5/6	Necrosis; new bone formation↓	ND	^117^
Rat	ND	M	ZA	ZA: 0.035	Every 15 days	TVI	8	4	The right max incisors	TE after the 4th dose	Approximately 9	BE(suppuration and bone sequestration); areas of osteolysis and fracture or loss of socket integrity	40%	^118^
Rat	16	M	ZA	ZA: 0.1	Weekly	SC	8	8	All left man molars	TE on the 7th week of drug administration	8	pseudo-epitheliomatous epithelium overlying exposed and/or unexposed bone with osteolytic lesions and clusters of EL	76.9%	^100^
Rat	9–10	ND	ZA	ZA: 0.1	At week 2 and 5	IV	5	2	The right man M1	TE on the 5th week (drug induction has finished)	13	Nonvital bone and EL; bone volume↓	100%	^101^
Rat	8	F	ZA	ZA: 0.1	Thrice a week	IP	9	27	The right max molars	TE after 9 weeks of drug administration	13/17	Osteonecrosis(10 adjacent EL)	13w: 83.3% 17w: 63.6%	^58^
Rat	13	F + M	ZA	ZA: 0.0075	Weekly	SC	11	11	Bilateral max M1	TE on the 3rd week of drug administration	11	Clinically exposed bone or a fistula; epithelium discontinuation with fragments of non-vital bone surrounded by non-specific inflammatory infiltrate	25%	^103^
			DEX	DEX: 1				11					0	
			ZA + DEX	ZA: 0.0075 DEX: 1				22					50%	
Mouse	8–12	F	ZA + CY	ZA: 0.05 CY: 150	ZA: Twice a week CY: Twice and once a week before and after tooth extraction	ZA: SC CY: IP	5	18	Max M1	TE after 3 weeks of drug administration	5	Open wounds; EL, living bone↓; the number of OCs↓	92.8%	^119^
Mouse	7–10	F	ZA	ZA: 0.1, 0.3, 0.5, 0.7 or 0.9	A bolus IV injection	IV	Once	1	The left max M1	TE after 1 week of drug administration	3	Abnormal oral mucosa swelling; osteonecrosis area↑	ND	^66^
Rat	10	F	ZA	ZA: 0.06	Weekly	TVI	2	2	The unilateral man M1	TE on the 2nd week of drug administration(1 wk after the first dose)	4/9	Discolored, brownish exposed bone, sometimes with accompanying pus discharge; small bone fragments suggestive of sequestra; EL	4w: 85.7% 9w: 57.1%	^120^
Rat	20	M	ZA	ZA: 0.06	Weekly	IV	6	7	The right man M1	TE after 1 week after the last drug administration	15	Extraoral signs of osteonecrosis; BE or fistula	78.3%	^70^
Rat	8	F	ZA	ZA: 0.08	Weekly	TVI	10	10	The right max M1, M2	TE after 2 weeks of drug administration	10	Exposed bone; BV/TV↓; open sockets with unhealed mucosa and the connective tissue collapsed; large amounts of NBs, empty bone lacunae; inflammatory cell infiltration and few OCs	61.5%	^71^
Rat	5	F	ZA + DEX	ZA: 0.0075 DEX: 7	ZA: 2/4/7 times within 14 days DEX: Everyday	SC	2	16/18/21	Three right molars	TE after the end of drug administration	4	Unhealed wound areas; ulcerated connective tissue; thin trabeculae, lined with multinuclear OCs; marrow spaces infiltrated with the inflammatory cells	2-ZA/DX: 20% 4/7-ZA/DX: 100%	^121^
Rat	5	M	ZA + DEX	ZA: 0.1 DEX: 1	Thrice a week	ZA: IP DEX: IM	10	60	Bilateral max M1	TE after 9 weeks of drug administration	10	Newly-formed woven bone inside the socket↓; areas of NB which were not lined by OCs; NB↑	ND	^72^
Rat	6–8	F	ZA + DEX	ZA: 0.2 DEX: 5	ZA: Weekly DEX: Thrice a week	ZA: TVI DEX: SC	8	32	The right max M1	TE after 8 weeks of drug administration	16	Incomplete mucosal healing and BE; destruction of cortical bone; the NB areas with EL	100%	^67^
Rat	12	F	ZA	ZA: 0.066	Thrice a week	IP	6/8/12	18/24/36	The right man and max M1	TE after 4 weeks of drug administration	6/8/12	BE; osteonecrosis (continued EL up to 5 in a row)	ND	^104^

*F* female; *M* male; *max* maxillary; *man* mandibular; *M1* first molar; *M2* second molar; *ND* data not found, *ref*. Reference

*TE* tooth extraction; *NB* necrotic bone; *BE* bone exposure; *OCs* osteoclasts; *EL* empty lacunae

*ZA* zoledronic acid/zoledronate; *DEX* dexamethasone; *mAb* rat anti-mouse *RANKL* monoclonal antibody; *CY* cyclophosphamide; *ALN* alendronate

*SC* subcutaneous injection; *IV* intravenous injection; *IP* intraperitoneal injection; *IM* intramuscular injection; *TVI* tail vein injection

*BV/TV* bone volume/tissue volume; *Tb.Sp* trabecular separation; *Tb.N* trabecular number; *Tb*.*Th* trabecular thickness; *BMD* bone mineral density

*TNF-α* tumor necrosis factor-α; *IL*-*1β* interleukin-1β; *TRAP* tartrate-resistant acid phosphatase; *TRAcP5b* TRAP isoform 5b; *β-TCP* β-tricalcium phosphate

Regarding species selection, approximately 60% of relevant studies chose rats rather than mice for research, usually 8–12 weeks old and female^44–48^. Rats were selected due to their low cost, rapid and easy reproduction, and simple maintenance conditions^49^. They have a larger size and can live longer in such a long experimental period compared with mice. Adult rodents were usually selected for studies based on clinical observation, suggesting osteonecrosis risk increased with age^17^. Yet mice are more advantageous for exploring biological mechanisms, especially transgenic mice^50^.

When it comes to drug administration for the systemic risk factor, antiresorptive drugs, mainly bisphosphonates (BPs) and anti-receptor activators of NF-κB ligand (RANKL) antibody (Ab) (denosumab in clinic), are the most common choice, especially BPs. In contrast, antiangiogenic drugs have not been widely used for general MRONJ models. RANKL monoclonal antibody (mAb) is usually given in combination with cyclophosphamide (CY) at a dose of mAb (5 mg·kg^−1^) once every three weeks plus CY (150 mg·kg^−1^) twice a week before tooth extraction and once a week after tooth extraction for five or seven weeks^51,52^. Among various BPs, ZA is the most potent induction drug because it is associated with the highest risk of MRONJ onset in clinic^53,54^. The induction dose of BPs on rodents is related to the dosage used in osteoporosis patients or bone tumor patients^17^. Kim et al. pointed out that the drug induction dose should refer to four aspects: the relevant ZA doses used in humans (oncologically, 67 µg·kg^−1^ for 4 weeks), the relatively rapid bone metabolism of rodents, the route of drug administration, and maximizing drug exposure during the relatively short experimental period^26^. Considering the routes of administration applied in MRONJ rodents, intraperitoneal administration is the simplest technique, but it requires a high drug dosage. Intravenous injection requires greater precision, but it possesses the fastest onset and the highest bioavailability—plasma drug concentrations of the subcutaneous and intramuscular routes are lower than those of the intraperitoneal and intravenous routes. In 2013, Kang et al. chose to use a higher dose of ZA, namely, 200 µg·kg^−1^, which is approximately three times the oncologic ZA dose, to increase the incidence of osteonecrosis in mice. They also elected to inject mice three times per week to mimic the monthly injections in humans, given the estimation that 17 days of a rodent’s life corresponds to one human year^41^.

In summary, the dosage of ZA ranges from 0.0075 to 2.25 mg·kg^−1^, with the most commonly used drug regimen being 0.1 mg·kg^−1^ ZA thrice a week for 6–9 weeks through intraperitoneal injection^47,55–58^. In addition, some studies used a corticosteroid drug such as DEX with BPs to increase the prevalence of osteonecrosis. Sanda et al. and other researchers created MRONJ models by subcutaneously or percutaneously administering ZA (0.066 mg·kg^−1^) and DEX (5 mg·kg^−1^) thrice a week for 4–6 weeks, and the success rate reached as high as 100%^46,59,60^.

The choice of extraction site varies less compared to drug induction. Most studies chose to remove the right or left maxillary first molar^59,61–67^. Maxillary teeth are easy to see during extraction, and compared with simultaneous extraction of bilateral maxillary first molars or unilateral three molars, the extraction of only one maxillary first molar is more straightforward, and it reduces the possibility of root fracture and causes minor trauma to the rodents, which is conducive to the rat’s recovery of feeding ability.

The time interval between tooth extraction and drug induction depends on the overall time arrangement of the model establishment. The priority of the time arrangement is to maximize drug exposure while relatively shortening the experimental period. The duration of drug induction varies in previous studies, but 4–6 weeks is most common. Almost all studies chose to administer ZA with or without DEX or other drugs for systemic induction lasting 2–4 weeks before tooth extraction. After tooth extraction, about half of the researchers administered medication until the end of the experiment, whereas the other half kept the rodents under persistent observation until MRONJ developed. Considering the clinical definition of MRONJ, bone exposure in the human maxillofacial region should persist for more than eight weeks, which corresponds to approximately one week of a rat’s life^55^. Thus, the MRONJ-like lesions should exist for at least one week in rodents before verifying the successful establishment of the MRONJ model. The total duration ranges from 2 weeks^68^ to 17 weeks^56,58^, although it exceeds eight weeks in most studies^31,44,45,69–72^, indicating a relatively long induction period.

In general, establishing the classical MRONJ rodent model is relatively simple, involving drug injection and tooth extraction. This approach has been widely used over the past two decades, suggesting good reliability and repeatability.

### Infection-inducing method

Classical methods of extracting healthy teeth fail to conform to current clinical practice, where tooth extraction often results from dental infectious diseases. Recent clinical studies have found that teeth that can be an infection source increase the risk of MRONJ, and tooth extraction itself maybe not be a risk factor^73–75^. Kim et al. put forward a model for MRONJ development with three hits: the first hit is a long-term medication history, the second is pathologic inflammatory conditions, and the third is structural defects in soft tissue integrity caused by dentoalveolar trauma^42^. This model attaches importance to associations between MRONJ occurrence and surgical interventions under pathologic inflammatory conditions. Thus, some researchers suggested inducing inflammation in the extraction site to better mimic tooth extraction based onset of MRONJ in clinic^19,37,42,76,77^.

Numerous rodent models under local dental infection (mainly pulpitis and periodontitis) have been established to complement classical methods by extracting infectious teeth. These modified methods adopting infection as a local risk factor can be summarized in three steps: injecting systemic drugs, inducing dental infection to create an inflammatory environment, and extracting the tooth in the inflamed area after a specified number of weeks^19,37,42,76,77^. Compared to the classical method, modified methods with infectious tooth extraction result in more severe MRONJ^19,42^. The presence of bone necrosis increases in infectious tooth extraction groups, with the larger necrotic bone areas and more empty osteocyte lacunae. Yet the overall time of establishing MRONJ is not prolonged compared with classical tooth extraction methods^19,42^.

As one of the hypotheses of MRONJ pathogenesis, infection is also linked with MRONJ without tooth extraction or other invasive procedures^14,78^. Thus, pure infection-induced MRONJ rodent models without tooth extraction are also an essential category of infection-induced models. Currently, MRONJ induced by infection is mainly divided into two categories: induction by pulpitis infection^19,37,39,79–81^ and induction by periodontitis infection^28,32,33,42,76,77,82–86^. We summarized methods adopting infection induction from the past three years in Table 2.

**Table 2:** Collection of MRONJ rodent models established by the infection-inducing method

Species	Age/ week	Sex	Drug dose/ (mg·kg^−1^)	Administration	Induction Time/week	Extraction site	Interval	MRONJ characteristics	Success rate	Year	Ref.
Mouse	11–12	F	ZA: 0.066 DEX: 5	ZA: thrice a week, IP DEX: weekly, SC	12	The left man M1	PE after 8 weeks of injection	Reduced periapical BL; noticeable and extensive areas of lacunae and osteocyte loss	20%	2019	^81^
Rat	8	M	ZA: 0.2	Twice a week, IP	9	The man M1, M2	PE after 1 week of injection; TE after 5 wks of injection	Delayed socket healing; reduced periapical BL; areas of lacunae and osteocyte loss	50%	2019	^37^
Rat	12	M	ZA: 0.2	Weekly, IP	8	The left man M1	PE after 3 weeks of injection	Intense zones of fibrosis and necrosis associated with acute inflammation	30%–60%	2018	^80^
Mouse	12	F	ZA: 0.066	Thrice a week, IP	12	The left man M1	PE after 8 weeks of injection	Areas of necrosis associated with the acute inflammatory process	ND	2017	^39^
Mouse	6	F	ZA: 125	Twice a week, IV	7	The left max M1	PE after 1 week of injection; TE after 4 weeks of injection	Prominent pulp exposure; histological presence of inflammatory cells and OCs	ND	2016	^19^
Mouse	16	M	RANK-Fc: 10 mg/kg; OPG-Fc:10 mg/kg	Thrice a week, IP	12	The right man M1, M2	PE after 3 weeks of injection	Prominent pulp exposure; histological presence of inflammatory cells and OCs	RANK-Fc: 10%; OPG-Fc: 10%	2014	^79^
Mouse	6	F	ZA: 0.125	Twice a week, IV	7	The max M2	Ligaturing after 1 week of injection; TE after 4 weeks of injection	EL and NB	ND	2020	^76^
Mouse	8	M	ZA: 0.2	Twice a week, IP	5	The right max M2	Ligaturing after 1 week of injection	EL and NB	ND	2019	^86^
Rat	12	F	ZA:0. 2	Weekly, IP	22	The left max M2	Ligaturing 12 weeks of injection	EL and NB	60%	2019	^32^
Mouse	6	F	ZA: 0.125	Twice a week, IV	4	The max M2	Ligaturing after 1 week of injection; TE after 4 weeks of injection	BL, NB, and EL	ND	2018	^42^
Rat	ND	ND	ZA: 0.2	Twice a week, IV	9	The max M2	Ligaturing after 1 week of injection; TE after 5 weeks of injection	BL, NB, and EL	100%	2018	^77^
Rat	12	F	ZA: 0.066	Thrice a week, IP	12	The left man M1	Ligaturing after 6 weeks of injection	No exposed NB but extensive EL.	ND	2016	^85^
Rat	12	F	ZA: 0.066	Thrice a week, IP	12	The left man M1	Ligaturing after 7 weeks of injection	Gingival recession and root exposure; no exposed NB.	ND	2015	^28^
Rice rat	4	F	ZA: 0.02–0.125	Every 4 weeks, IV	12/18/24/30	None	None	BL, NB, and EL	Gross MRONJ:22%; histologic MRONJ:73%	2017	^33^
Rice rat	4	M	ZA: 0.08	Every 4 weeks, IV	24	None	None	BL, NB, and EL	50%	2021	^82^
Rice rat	4	M	ZA: 0.08	Every 4 weeks, IV	24	None	None	BL, NB, and EL	50%	2020	^83^
Rice rat	4	F	ZA: 0.02–0.125	Every 4 weeks, IV	12/18/24/30	None	None	BL, NB, and EL	Gross MRONJ:18%; histologic MRONJ:35%	2019	^84^

*F* female; *M* male; *max* maxillary; *man* mandibular; *M1* first molar; *M2* second molar; *ND* no data found; *ref*. reference;

*TE* tooth extraction; *NB* necrotic bone; *EL* empty lacunae; *BL* bone loss; *OCs* osteoclasts; *PE* pulp exposure

*ZA* zoledronic acid/zoledronate; *DEX* dexamethasone; *RANK-Fc* composed of the extracellular domain of RANK fused to the fragment crystallizable [Fc] portion of immunoglobulin G [IgG]); *OPG-Fc*: composed of the RANKL-binding domains of osteoprotegerin [OPG] linked to the Fc portion of IgG

*SC* subcutaneous injection; *IV* intravenous injection; *IP* intraperitoneal injection

Generally, MRONJ induction by pulpitis involves drilling a hole in the target tooth (first and second molars) to expose pulp for at least three weeks. The time of systemic drug induction is more than eight weeks. MRONJ induction by periodontitis, as another popular infection method, typically involves ligaturing^28,32,42,76,77,85,86^ or a high sugar diet without ligaturing in rice rat models^33,82–84^. Ligaturing is the conventional method for periodontitis induction; as shown in Table 2, more than half of the studies using MRONJ induction by periodontitis adopted ligaturing. The general process of ligaturing is similar to pulp exposure: silk ligatures are wrapped around the neck of the target tooth for at least 3 weeks of periodontitis infection, and the overall time of systemic drug induction is over four weeks.

The eventual assessment of pure infection-induced MRONJ rodent models without tooth extraction is generally regarded as MRONJ stage 0. There is no visible epithelial damage or necrotic bone but there is pathological necrotic bone and empty bone lacunae. This could be due to the lack of the third hit in the MRONJ development model^42^, because structural defects in soft tissue integrity are caused by tooth extraction. Several studies have tried to improve induction methods to generate more obvious MRONJ symptoms. As MRONJ development is associated with induction time, Hadaya et al. extended the ligaturing time to 10 weeks and the overall administration time to 22 weeks; the MRONJ model tissue sections showed continuous epithelial damage and necrotic bone exposure^32^.

As a standard animal periodontitis model, rice rats have also been used as MRONJ models with generalized periodontitis induced by a high sugar diet without ligaturing^33,82–84^. The occurrence of gross MRONJ with exposure to the alveolar or palatal bone in rice rats is 13%–18%, and histological MRONJ is around 70%^33,84^. The systemic drug induction of rice rats is different from rats and mice. The most commonly used dose of ZA on rice rats is 80 μg·kg^−1^, injected every 4 weeks for 24 weeks^82,83^. Compared to rat or mouse models, the induction time of rice rats is significantly prolonged. But the induction method is simplified as a standard diet with reduced ZA administration frequency.

Pulpitis and periodontitis are bacterial infections, and bone exposure to the oral cavity provides access to oral bacterial invasion in MRONJ development. As some studies pay attention to the function of oral microbiota colonized on the bone surface of MRONJ^28,87^, it is worth noting that to better control the baseline, amoxicillin, metronidazole, and other antibiotics are often used to remove possible oral pathogens ahead of exerting stimulation. Furthermore, a wash-away period of about 3 days is used to eliminate the impact of indigenous antibiotics^76^.

### Mechanical stimuli-inducing method

Mechanical stimuli besides tooth extraction have also been adopted as local risk factors in establishing MRONJ models, such as implantation, because the clinical risk of MRONJ onset after implantation is comparable to that after tooth extraction^1^. Inoue et al. placed an implant in rat maxilla after 12 weeks of drug injection to mimic the development of MRONJ around implants^88^. Bone grinding by drilling has also been used for more significant bone defects in establishing MRONJ models^89^. In addition to invasive dental procedures, other factors which exert sustained and micromechanical stimuli may also induce MRONJ. Previous studies have reported that occlusal overload on the prosthesis or caused by rheumatoid arthritis might have contributed to MRONJ^90,91^, but methods for establishing MRONJ models based on sustained mechanical stimuli are still in the preliminary stages. Mine et al. developed a novel mouse model with experimental hyperocclusion to investigate the potential role of occlusal/mechanical trauma in MRONJ^92^. This model provides reasonable evidence for the feasibility of using the mechanical load in MRONJ models. However, the specific role of mechanical stimuli in MRONJ development has not been verified.

**Fig. 3:**
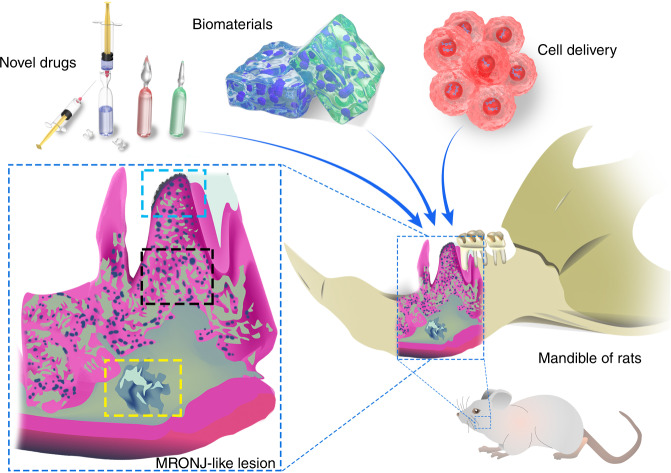
Main approaches for MRONJ treatment tested in rodent models involve novel drugs, biomaterials, and gene-engineered cells for delivery. A typical MRONJ mandible presents a prominent gingival ulcer with exposed necrotic bone (blue), osteolysis (black), and abscess formation (yellow)

In short, classical methods, which combine systemic drug injection with healthy tooth extraction, currently offer the most versatility for MRONJ research. Modified methods adopting infection induction are expected to improve upon classical methods by extracting infectious teeth, thereby mimicking tooth extraction resulting from dental infections, which is much closer to MRONJ onset in clinic. Some MRONJ models are established by implantation, trauma, hyperocclusion, and other mechanical stimuli. For long-term MRONJ progression, we suggest that for research on the pathogenesis of MRONJ, various time points can be set in the pretest study to collect samples and examine the occurrence and development of MRONJ no matter which local risk factors are adopted. As an in vivo experimental platform, the timing of exerting preventive and therapeutic interventions on MRONJ rodent models should also be considered. For research on MRONJ prevention, interventions can be exerted immediately after applying local risk factors to observe whether the experimental manipulation can delay or inhibit the occurrence of MRONJ. Before testing the efficacy of interventions on treatments, it is recommended to wait for the completion of MRONJ models with a systematic evaluation, which verifies the selected animals have been successfully modeled for follow-up experiments, generally including tests on newly developed drugs and diverse applications of existing drugs, biological materials, and cell delivery (Fig. 3).

## Assessment of MRONJ-like lesions in rodent models

Assessment of MRONJ-like lesions in rodent models provides evidence presenting the effectiveness and efficiency of adopted methods. The assessment is generally composed of two parts: the occurrence rate of MRONJ and the characteristics of MRONJ-like lesions. Judgment on the occurrence of MRONJ includes the onset of gross MRONJ and histological MRONJ. The gross MRONJ presents unhealing mucosa and exposed bone observable by the naked eye. The definition of histological MRONJ varies among previous studies, but necrotic bone is the gold standard. It is thus clear that assessment of occurrence is based on evaluating characteristics of MRONJ-like lesions in rodents. As various techniques have been introduced to assess MRONJ-like lesions, we comprehensively overviewed current practices in gross observation, histological assessments, radiographic assessments, and serological assessments in Table 3. Generally, gross observation and histological and radiographic assessments have three essential aspects for assessing MRONJ rodents’ lesions. First, all the studies presented histological and pathological findings. Second, more than half showed gross observation by the naked eye or radiographic assessment. Third, indicators for quantitative analysis have also been widely used.

**Table 3:** Assessment system of MRONJ-like lesions

Aspects	Indicators	Illustration
Gross observations	Soft tissue^30,44,45,47,62,64,67,72,93–96^	Indicated by the color, texture, and integrity of oral mucosa.
	Bone exposure^30,44,45,47,62,64,67,72,93–96^	Indicated by the area, time of bone exposure.
Histopathological assessments	Healing conditions^59,60,63,98^	Histological sections show the soft tissue healing with the distance between the edges of the epithelia, and bone defects with the length of the necrotic bone exposed towards the oral cavity.
	Necrotic bone^49,57–59,64,69,72,96,100^	The presence of necrotic bone represents the occurrence of MRONJ. The definition of necrotic bone depends on the number of confluent empty or karyolytic osteocytic lacunae.
	Empty bone lacunae^30,45,95,96,98^	The proportion or the number of empty bone lacunae in a certain area indicates the degree of osteocyte loss, which present the bone necrosis.
	TRAP^+^ osteoclast^44,49,57,58,64,67,69,72,93,95–101^	The TRAP^+^ osteoclasts present the bone resorption, commonly used indicators including numbers of osteoclast per area or per bone line.
	Osteoblast^30,44,49,64,67,93,97^	Generally identified by hematoxylin-eosin staining or marked by alkaline phosphatase (ALP), bone morphogenetic protein-2 (BMP-2), or receptor activators of NF-κB ligand (RANKL), commonly used indicators including numbers of osteoclast per area or per bone line.
	Blood vessels^65,67,69^	The extent of angiogenic inhibition is assessed by the density of blood vessels generally marked by CD31.
	Inflammation^49,67,99^	The extent of inflammation is assessed by the number of polymorphonuclear cells under fixed area, as well as the infiltration and bone sequestra.
Serological assessments	VEGF^67,98,103^	Serum VEGF presents the angiogenic ability of MRONJ.
	GluOC^64^/CTX-1^98^/TRAcP-5b^64^/P1NP^64^	Bone metabolism markers of MRONJ under further exploration.
Radiographic assessments	μCT	To present bone healing conditions, bone sequestra formation of MRONJ, with parameters of bone volume/tissue volume^30,48,59,60,69,95–98,100,101,103,104^, trabecular separation^69,96,98,100,104^, trabecular thickness, trabecular number and bone volume/tissue volume representing bone morphological markers related to the early stage of MRONJ.
	PET/CT^108^	To present bone metabolism and inflammation with specificity and higher resolution.
	Portable X-ray devices^94^	To present bone quality by drawing the Regions of interest (ROI) to obtain the attenuation coefficient (similar to BV/TV), the ratio between the average ROI values on the surgery side and the control side.
	SEM^101^	To present osteocytes in bone lacunae.
	TEM^106^	To illustrate osteoclasts with ruffled border adjacent to the alveolar wall.
	Raman spectroscopy^47^	To calculate mineral/matrix ratio and carbonate/phosphate.
	ICG-based NIF imaging^89^	To mark affected bone tissues with pathological examination with quantification detection of fluorescence intensity.
	A cross-modality imaging pipeline^107^	To combine Atomic Force Microscopy and Scanning Electron Microscopy to acquire complementary hallmarks of MRONJ.

*MRONJ* medication-related osteonecrosis of the jaw; *TRAP* tartrate-resistant acid phosphatase; *VEGF* vascular endothelial growth factor; *GluOC* uncarboxylated osteocalcin; *CTX-1* C-terminal peptide of type I collagen; *TRAcP-5b* tartrate-resistant acid phosphatase 5b; *P1NP* N-terminal propeptide of type I procollagen; *PET/CT* Positron emission tomography/computed tomography; *μCT* micro-computed tomography; *SEM* scanning electron microscope; *TEM* transmission electron microscope; *ICG* indocyanine green; *NIF* near-infrared fluorescence; *PET/CT* positron emission tomography/computed tomography

In addition to the comprehensive assessment system, we also highlighted the critical aspects of assessment. However, the heterogeneity of methods for establishing MRONJ rodent models in terms of species, drug type, and local risk factors brings difficulty to finding general characteristics in assessing MRONJ-like lesions. To reduce the bias in the evaluation, we screened the past 3 years of studies on establishing rat models by classical methods from Table 1. Also, we took into consideration other studies that utilized novel detection approaches.

### Gross observation

Mucosal healing and bone exposure are two important indexes in gross observation. Most studies mainly describe the gross MRONJ as “incomplete and delayed mucosal healing and bone exposure”^30,44,45,47,62,64,67,72,93–96^. However, in infection-induced MRONJ rodent models, the gross observation may only present mild to moderate inflammation with soft tissue swelling and erythema without exposure to bone tissue on probing^45,85^. For pulp-exposed MRONJ rodent models, there may even be no oral mucosal lesion or exposed bone observed^39^.

Some studies conducted quantitative analysis; the whole unhealed mucosal area and ratios of the exposed area were calculated to assess the wound healing conditions^97,98^. Different scoring systems of mucosal healing were also put forward by researchers^67,72^. Merloni et al. defined three stages of healing conditions by the exposed area ratio as grade 1: socket surface more comprehensive than the occlusal area of the second maxillary molar and dark, rough, and irregular appearance of the wound surface; grade 2: intermediate features between grades 1 and 3; and grade 3: socket surface more minor than the occlusal area of the second maxillary molar^72^. Gao et al. established a blind healing score with ten levels based on the degree of mucosal closure in gross observation and the detection of sequestration in radiographic images^67^. The details of the healing score are as follows: 1–3: exposed necrotic bone plus evidence of sequestration in the micro-computed tomography (μCT); 4–6: mixed granulation tissue and mucosal coverage, some exposure, and no evidence of sequestration in μCT; 7–9: mixed granulation tissue and mucosal coverage with complete coverage, no sequestration; and 10: normal mucosal coverage, no sequestration, evidence of the regular bone filling of the defect^67^. For rice rats, the assessment of gross MRONJ depends on gross quadrant grade (GQG) (0–4), showing the severity of soft tissue lesions. Gross MRONJ of rice rat is defined as a lesion of GQG = 3 or 4 with exposure of alveolar or palatal bone^33,84^. It should be noted that the same calibrated examiner should perform the assignment of the score and measurement in a blind fashion.

### Histological assessments

The indicators in histological sections commonly used to assess the healing conditions are epithelial integrity, inflammation, bone necrosis, osteoclast, osteoblast, and blood vessels. In histological assessment, the epithelial integrity usually refers to the length of necrotic bone exposed toward the oral cavity and the distance between the edges of the epithelial surfaces^59,60,63,98^. The distance between the edges of the epithelia is defined as the shortest end-to-end distance of the ripped epithelium^60^. The length of the necrotic bone exposed toward the oral cavity is defined as the longest distance of the exposed necrotic bone^60,63^. This assessment should note the consistency of the placement of samples when embedded in paraffin. The method of Soundia et al., useful as a reference, involves making cross-sections perpendicular to the long axis of the alveolar ridge at the area of the mucosal defect or the area between the first and second molars to determine whether the mucosa is complete healed^97^.

Inflammatory conditions are also assessed. The simplest method for inflammation evaluation is to use the number of polymorphonuclear cells under a fixed area^49,67,99^. Some studies utilized a simple scoring system based on the intensity of inflammatory cells in the defect area as 0: no inflammation; 1: mild inflammation; 2: moderate inflammation; and 3: severe inflammation^49,57,93^. A more complex system further considers the infiltration and bone sequestra for a comprehensive assessment^63^.

The formation of necrotic bone is the most important hallmark of MRONJ occurrence in rodent models. However, the definition of necrotic bone differs among various studies^49,57–59,64,69,72,96,100^. Generally, necrotic bone is identified as an area of at least 5–10 confluent empty or karyolytic osteocyte lacunae^49,57–59,69,72,96,100^. The area of necrotic bone was defined as the bone area with empty osteocyte lacunae, and the necrotic bone ratio was defined as the ratio of empty occupied osteocyte lacunae^59,60^. Instead of measuring necrotic bone, some studies directly used the average number of empty lacunae per area to indicate bone necrosis^30,45,95,96,98^. According to the histology images and statistics presented in different studies, it should be more objective and persuasive to calculate the empty lacunae and analyze the necrotic bone area to reflect the overall situation of bone defects.

The cell number/density of osteoblasts or osteoclasts is an essential indicator in histological assessment to reflect MRONJ changes at the cell level. The attention to osteoblasts is lower than osteoclasts, as among 34 studies adopting ZA-treated rats selected from Table 1, seven presented osteoblast statistics^30,44,49,64,67,93,97^, whereas over half presented osteoclast statistics^44,49,57,58,64,67,69,72,93,95–101^. The attached osteoclasts were counted per linear bone perimeter using tartrate-resistant acid phosphatase (TRAP) staining. Although antiresorptive drugs suppress the activity of osteoclasts, the density of detached osteoclasts and attached osteoclasts increases in MRONJ-like lesions^30,62^. For standardization comparison, it is recommended to calculate the number of osteoclasts or osteoblasts with the length of the bone surface.

Disturbance in blood vessels is also one of the pathological characteristics of MRONJ. Studies have found that sustained ZA treatment causes a microcirculatory inflammatory reaction in the mandibular periosteum^102^. Thus, vessel assessment was performed mainly in studies exploring angiogenic effects in MRONJ models^65,72^. CD31 is the most commonly used marker, indicating vessels in histological sections^65,67,69^. Tamari et al. creatively utilized Dil molecules directly incorporated into the cell membrane, labeling functional vessels in red^103^.

### Radiographic assessments

Radiographic methods contribute to evaluating bone quality and formation of bone sequestra in MRONJ defects. Micro-CT (µCT) imaging was the most commonly used method. The description of MRONJ sites is usually based on sectional images or 3D stereoscopic images constructed from μCT data. For quantitative statistics, bone morphometric indices of bone volume fraction and bone volume/tissue volume (BV/TV) were utilized most frequently^30,48,59,60,69,95–98,100,101,103,104^, which generally showed a significant decrease in most rat models^30,48,60,69,95–97,100,101,103,104^. Trabecular separation (Tb.Sp) is another parameter from µCT, presenting thickness of space, in which a higher value indicates reduced connectivity of trabecular bone. Tb.Sp of MRONJ rodents shows an increasing trend^69,96,98,100,104^. Bone mineral density (BMD) exhibiting the bone mineral mass per bone volume was also calculated to assess the newly formed bone in MRONJ models used for testing therapeutic interventions^69,95,104^. 3D images constructed by μCT were also adopted to evaluate the size of bone defects comprehensively^105^.

In addition to µCT examination, other radiographic methods have been applied for assessing the bone quality of MRONJ-like lesions in rodents. Paulo et al. used a portable X-ray device for radiographic evaluation and analyzed the images through ImageJ^94^. To obtain the attenuation coefficient (similar to BV/TV), the ratio between the average values on the surgery side of the mandible and the control side was calculated. To assess changes in bone composition, De Sousa Ferreira et al. adopted Raman spectroscopy to calculate mineral/matrix ratio in bone tissue^47^. Besides, a scanning electron microscope (SEM) and a transmission electron microscope (TEM) were utilized for optical imaging to assess the cell morphology of osteoblasts^101^ and osteoclasts^106^. Reier et al. established a cross-modality imaging pipeline combining µCT with atomic force microscopy and SEM to acquire complementary hallmarks of MRONJ^107^. These radiographic methods present changes in bone quality and cell morphology in MRONJ-like lesions, which is beneficial for further exploration of MRONJ pathophysiology.

Novel radiographic methods for MRONJ diagnosis and treatment have been tested in MRONJ rodent models to explore their feasibility in clinic. Positron emission tomography/computed tomography (PET/CT) appeared to be a sensitive imaging modality for identifying markers of inflammation and bone metabolism to diagnose MRONJ in a rat model, including a ZA/DEX group^108^. The decreased bone remodeling tendency highlighted by PET/CT may indicate a possible risk of MRONJ before the onset of clinical signs and symptoms. Xia et al. utilized indocyanine green (ICG), a molecular probe applied in bio-imaging for many years, to mark MRONJ-affected bone for removal and preserve normal tissue as much as possible for the first time in a rat model^89^. Applying these radiographic methods in MRONJ rodent models has generated preclinical evidence in support of their feasibility for diagnosis and treatment.

### Serological assessments

MRONJ presents disorders in bone turnover, which results in changes in bone formation and resorption products. Detection of these characteristic products in serum can be a promising approach to the prediction and treatment of MRONJ. Although serological assessment is not a general examination in rodent models, attention to serum markers for MRONJ has increased^64,67,98,103^. A decrease in the statistical significance of serum vascular endothelial growth factor (VEGF) was found in an MRONJ group^67,98,103^, which indicated the inhibition of angiogenesis. Serological bone turnover indicators such as uncarboxylated osteocalcin (GluOC)^64^, C-terminal peptide of type I collagen (CTX-1)^98^, tartrate-resistant acid phosphatase 5b (TRAcP-5b)^64^, and N-terminal propeptide of type I procollagen (P1NP)^64^ were also detected in the MRONJ assessment. However, these bone turnover indicators are still controversial as biomarkers of MRONJ^109,110^. The significance of bone metabolism markers of MRONJ still needs further preclinical and clinical evidence.

## Conclusion

Methods for establishing MRONJ rodent models have evolved as the understanding of MRONJ pathogenesis, especially local risk factors, has deepened. Tooth extraction is the most commonly used local risk factor, which assumes a central role in exploring pathogenesis and testing novel interventions. Various approaches to infection induction have also been developed to better mimic MRONJ onset following patients’ clinical status. Mechanical stimuli have emerged in model establishment, including implantation and other invasive procedures and sustained stress from hyperocclusion. Modified methods with the extraction of infectious teeth are expected to become superior alternatives to classical methods as they present more obvious MRONJ-like lesions that conform more closely to lesions encountered in clinical practice.

Because there is still a lack of a standard assessment system for MRONJ rodent models, we summarized current techniques for assessing MRONJ-like lesions. The histological assessment is the most effective method, mainly characterized by empty osteocyte lacunae. Meanwhile, gross observation, radiographic assessment, and serum indicators also contribute to the comprehensive MRONJ-like lesion examination. Although MRONJ rodent models are gradually becoming more mature and reliable with more comprehensive assessment criteria, establishing models simulating natural MRONJ pathogenesis is still challenging due to the long induction time and onerous induction procedures. Shortening the induction time with an improved success rate is critical for future research on MRONJ rodent models because of its lower cost and higher efficacy. Emerging approaches such as new-found risk factors and distinctive drug combinations have sprung up in MRONJ-related studies, which are expected to improve the establishment of MRONJ rodent models. Researchers still need to continue exploring how to more comprehensively simulate the clinical pathogenesis of MRONJ to make rodent models more reliable for preclinical research.
